# Serum PCSK6 and corin levels are not associated with cardiovascular outcomes in patients undergoing coronary angiography

**DOI:** 10.1371/journal.pone.0226129

**Published:** 2019-12-11

**Authors:** Shang-Feng Yang, Ruey-Hsing Chou, Shing-Jong Lin, Szu-Yuan Li, Po-Hsun Huang

**Affiliations:** 1 Division of Nephrology, Department of Medicine, Cheng Hsin General Hospital, Taipei, Taiwan; 2 Institute of Clinical Medicine, National Yang-Ming University, Taipei, Taiwan; 3 Division of Cardiology, Department of Medicine, Taipei Veterans General Hospital, Taipei, Taiwan; 4 Department of Critical Care Medicine, Taipei Veterans General Hospital, Taipei, Taiwan; 5 Healthcare and Management Center, Taipei Veterans General Hospital, Taipei, Taiwan; 6 Taipei Heart Institute, Taipei Medical University, Taipei, Taiwan; 7 Division of Nephrology, Department of Medicine, Taipei Veterans General Hospital, Taipei, Taiwan; 8 Cardiovascular Research Center, Taipei Veterans General Hospital, Taipei, Taiwan; Erasmus Medical Center, NETHERLANDS

## Abstract

**Introduction:**

Proprotein convertase subtilisin/kexin-6 (PCSK6) is a secretory protein that activates corin in the heart. Higher circulating levels of corin are associated with improved cardiovascular outcomes in patients with acute myocardial infarction. This study aimed to determine the role of serum PCSK6 and corin levels in predicting cardiovascular outcomes in patients with suspected coronary artery disease (CAD).

**Materials and methods:**

In total, 565 patients who had undergone coronary angiography were enrolled. Serum PCSK6 and corin levels were determined before the administration of contrast media. In this study, coronary revascularization, acute myocardial infarction, acute stroke, and death were defined as cardiovascular outcomes. All patients were followed up for at least one year after coronary angiography or until the occurrence of death.

**Results:**

During a median follow-up of 691 days, 67 patients (15.7%) developed composite cardiovascular outcomes after coronary angiography, including 51 incidents of coronary revascularization, 7 instances of acute myocardial infarction, 2 acute strokes, and 15 deaths. After adjustment for demographic characteristics and all significant variables in the univariate analysis, serum levels of neither PCSK6 nor corin were associated with increased risk for cardiovascular outcomes. This correlation remained insignificant in patients with underlying hypertension, diabetes mellitus, CAD, heart failure, or chronic kidney disease (CKD). However, in patients without CKD, higher serum PCSK6 levels were associated with increased risk for cardiovascular outcomes (hazard ratio 1.380; 95% confidence interval 1.023–1.862).

**Conclusions:**

We found no association between cardiovascular outcomes and pre-procedural serum levels of PCSK6 or corin in patients undergoing coronary angiography. However, an increased risk was seen in non-CKD patients with higher PCSK6 levels. Further studies are needed to verify these results.

## Introduction

Corin is a type II transmembrane serine protease that is highly expressed in atrial cardiomyocytes [1]. Corin is a mosaic protein consisting of a transmembrane domain near the N terminus and two frizzled-like domains, eight low-density lipoprotein (LDL) receptor repeats, a scavenger receptor-like domain, and a trypsin-like protease domain at the C terminus [2, 3]. Corin plays a significant role in converting pro-atrial natriuretic peptide (pro-ANP) to its active form, ANP. Previous studies have shown that cardiac corin expression is upregulated under pathological conditions and that corin over-expression was associated with improved outcomes in an animal model of heart failure [4, 5]. In humans, lower corin activity is associated with higher risk for hypertension, chronic heart failure, and/or cardiac mortality [6–8]. Therefore, corin-mediated natriuretic activation is essential for the maintenance of normal blood pressure and cardiac function.

Corin is made as a zymogen, which is activated by proprotein convertase subtilisin/kexin-6 (PCSK6) at a conserved site, Arg801-Ile802 [9, 10]. PCSK6 is a member of the proprotein convertase family and is expressed in many tissues, including muscle, heart, pituitary, intestine, cerebellum, and kidney [11]. PCSK6 was recently found to be secreted by cardiomyocytes and to activate corin on the cell surface [9, 10]. In previous studies, decreased PCSK6 activity was associated with a hypertensive phenotype in PCSK6-knockout mice and in humans [12]. These results suggest that PCSK6 may influence the regulation of blood pressure through downstream activation of corin and ANP.

Various isoforms of corin can be detected in the circulation because of ectodomain shedding [2, 13]. Recent studies demonstrated a prognostic role for circulatory corin in relation to various cardiovascular outcomes in patients with acute myocardial infarction (AMI), chronic heart failure, and acute stroke [14–17]. However, whether serum PCSK6 levels may be used to predict cardiovascular outcomes in patients with suspected coronary artery disease (CAD) remains uncertain. Therefore, we hypothesized that serum PCSK6 and corin levels may predict cardiovascular outcomes in patients with suspected CAD. In this study, we evaluated the relationship between serum PCSK6 and corin levels and the incidence of cardiovascular outcomes in patients undergoing coronary angiography.

## Materials and methods

### Study population

Detailed materials and methods had been described in our previous work [18]. In short, 565 consecutive patients who were admitted to a single tertiary medical center for coronary angiography between December 2009 and February 2015 were evaluated. Coronary angiography was indicated for patients presented to cardiovascular outpatient clinic with typical symptoms of angina and positive findings of treadmill exam or thallium perfusion scan. Informed consents were obtained on admission and blood samples were collected before coronary angiography. One hundred and seven subjects were excluded due to lost to follow-up and 32 individuals were excluded due to end stage renal disease (ESRD), which was defined as an estimated glomerular filtration rate (eGFR) < 15 mL/min/1.73 m^2^ or pre-existing dialysis,. Ultimately, a total of 426 subjects were enrolled in this study. Before enrollment, subject’s data on medications, smoking status, and risk factors for cardiovascular disease such as age, hypertension, type 2 diabetes mellitus, heart failure, and CKD were reviewed in detail. Blood pressure measurements were performed with electronic sphygmomanometers on the day of coronary angiography. Hypertension was defined as systolic blood pressure ≥ 140 mmHg, diastolic blood pressure ≥ 90 mmHg, or use of antihypertensive medications. Type 2 diabetes mellitus was defined as fasting plasma glucose ≥ 126 mg/dL or use of hypoglycemic agents. CKD was defined as eGFR < 60 mL/min/1.73 m^2^. eGFR was calculated using age, sex, and serum levels of blood urea nitrogen, creatinine, and albumin, according to the modified equations used to estimate glomerular filtration rate in Chinese patients [19]. Body mass index (BMI) was calculated by dividing the weight of the patient in kilograms by the square of the height in meters. Nonionic low-osmolality contrast media (iopromide) was administered intra-arterially, mainly through transradial catheters. Physiological (0.9%) saline was given intravenously at a rate of 1 mL/kg/h for 12 hr before and after contrast media exposure. The hydration rate was reduced to 0.5 mL/kg/h for patients with left ventricular ejection fraction < 40% or apparent heart failure. The study protocol was approved by the institutional review board of Taipei Veterans General Hospital. Written informed consent was obtained from all participants, and our study complies with the Declaration of Helsinki.

### Laboratory investigations

Blood samples were obtained from each patient after ≥ 8 hr of fasting on the date of coronary angiography. Serum levels of uric acid and glucose were measured using a Hitachi 7600 Autoanalyzer (Hitachi Ltd., Tokyo, Japan). Urine dipstick analysis was performed with a commercial test strip. Proteinuria was defined as urine protein ≥ 30 mg per 100 mL in urinalysis. Serum concentrations of PCSK6 and corin were determined with a commercial enzyme-linked immunosorbent assay (ELISA) (MyBioSource and R&D Systems, Inc.). Sensitivity to PCSK6 was 5 ng/mL. Sensitivity to corin was 7 pg/mL. Patients were classified into two groups according to serum PCSK6 levels. Subjects with PCSK6 concentrations higher than the median were labeled as the “high PCSK6 group”; all others were included in the “low PCSK6 group”.

### End-points for clinical follow-up

Patients were advised to visit outpatient clinics regularly after discharge from the hospital. The cohort was followed until January 2016. Patients’ clinical data were obtained every 3–6 months during follow-up. The composite cardiovascular (CV) outcome was defined as coronary revascularization, acute myocardial infarction, acute stroke, and/or death.

### Statistical analysis

Data are expressed as median (quartiles) for numeric variables and as number (percent) for categorical variables. Clinical and laboratory data were compared using the Mann-Whitney U-test for continuous variables and Fisher’s exact test for categorical variables. Logarithmic (log) transformation was performed to achieve a normal distribution for skewed variables (PCSK6, corin, and eGFR). The incidence of the composite CV outcome described above was calculated. Survival curves were generated with the Kaplan–Meier method, and survival among groups was compared by log-rank test. Cox proportional hazard regression analysis was performed to investigate the risk factors for composite CV outcomes. Factors with statistical significance in univariate regression analysis were entered into a final forward stepwise multivariate logistic regression model.

Because of the protective role of natriuretic peptide in cardiac remodeling under stress, we hypothesized that the prognostic value of serum PCSK6 and corin may be more significant in patients with higher cardiovascular risk. We performed a subgroup analysis and stratified the study cohort by the presence of hypertension, diabetes mellitus, CAD, heart failure, and CKD. We also performed linear regression analysis to investigate the possible correlation between the serum PCSK6 and corin levels. Data were analyzed using SPSS version 18.0 (SPSS Inc., Chicago, IL). A *p*-value < 0.05 was regarded as statistically significant.

## Results

### Baseline characteristics

The median age of the study population was 71 years (interquartile range, IQR, 60–81), and 69.5% were male. Table 1 summarizes the baseline characteristics of patients, grouped according to serum PCSK6 concentrations. There were no differences between patients in the low PCSK6 group and those in the high PCSK6 group with respect to BMI; medical history; use of antiplatelet agents, beta-blockers, or statins; serum levels of hemoglobin; fasting glucose, LDL, or high-density lipoprotein levels; eGFR; levels of uric acid, proteinuria, or corin; risk for diagnosis with CAD; or syntax score, as determined by coronary angiography. Subjects with higher serum PCSK6 concentrations tended to be younger (median, 69 years; IQR 57–80) and to use ACE-I or ARB (21.1%, n = 45). Subjects with higher serum PCSK6 were also less likely to be male (64.3%, n = 137) and current smokers (33.8%, n = 72). In multivariate linear regression analysis (S1 Table), serum PCSK6 levels were negatively correlated with age (β = -0.127, p = 0.015) and with male gender (β = -0.120, p = 0.022).

**Table 1:** Baseline patient characteristics, stratified by median level of serum PCSK6.

Characteristic	Total	PCSK6 < 56.8 ng/mL	PCSK6 ≥ 56.8 ng/mL	*P* value
	n = 426	n = 213	n = 213	
Age (years)	71 (60–81)	73 (62–81)	69 (57–80)	0.018
Sex (male)	296 (69.5)	159 (75.6)	137 (64.3)	0.013
Smoking	164 (38.5)	92 (43.2)	72 (33.8)	0.029
BMI (kg/m^2^)	25.38 (23.1–27.9)	25.5 (23.5–27.9)	25.1 (22.8–27.9)	0.375
Medical History				
Hypertension	297 (69.7)	155 (72.8)	142 (66.7)	0.103
Diabetes mellitus	160 (37.6)	87 (40.8)	73 (34.3)	0.097
Heart failure	75 (17.6)	37 (17.4)	38 (17.8)	0.500
Chronic kidney disease	115 (27.0)	59 (27.7)	56 (26.3)	0.414
Medications				
Antiplatelet	251 (58.9)	135 (63.4)	116 (54.4)	0.076
ACEi or ARB	107 (25.1)	62 (29.1)	45 (21.1)	0.037
BB	93 (21.8)	51 (23.9)	42 (19.7)	0.348
Statin	114 (26.8)	63 (29.6)	51 (24.0)	0.114
Laboratory data				
Hemoglobin (g/dL)	12.9 (11.6–13.9)	12.9 (11.6–13.9)	13 (11.6–14)	0.372
Fasting glucose (mg/dL)	105 (93–125)	107 (93–128)	104 (92–122.5)	0.361
Low density lipoprotein (mg/dL)	96.5 (76–113)	93 (75–109)	96.5 (76.5–115)	0.104
High density lipoprotein (mg/dL)	41.8 (31.7–54)	41.6 (31.8–52.6)	42 (31.7–55.1)	0.966
eGFR (mL/min/1.73 m^2^)	76.1 (57.3–94)	74.7 (57–88.3)	78.7 (58.4–97.2)	0.082
Uric acid (mg/dL)	6.1 (4.8–7.2)	6.1 (6.1–7.2)	6.1 (4.8–7.2)	0.876
Proteinuria, n (%)	59 (13.8)	27 (12.7)	32 (15.0)	0.288
PCSK6 (ng/mL)	56.4 (23.4–159.0)	24.4 (10.4–36.0)	158.2 (87.2–384.7)	<0.001
Corin (pg/mL)	1047.9 (746.4–1361.5)	1062.8 (788.2–1360.9)	1040.3 (710.9–1388.1)	0.619
Coronary angiography				
Coronary artery disease	242 (56.8)	128 (60.1)	114 (53.5)	0.102
Syntax score	3 (0–14)	5 (0–14)	2 (0–12.5)	0.239

Data are presented as median (interquartile range) or as total number of patients (%).

BMI, body mass index; ACEi, angiotensin-converting enzyme inhibitor; ARB, angiotensin II receptor blocker; eGFR, estimated glomerular filtration rate

### Independent predictors of composite CV outcomes

All patients were successfully followed up for a median duration of 691 days (IQR, 441–1105 days). Sixty-seven patients (15.7%) developed composite CV outcomes after coronary angiography, including 51 instances of coronary revascularization, 7 acute myocardial infarctions, 2 acute strokes, and 15 deaths.

The incidence of composite CV outcomes was similar between patients with higher vs. lower serum PCSK6 concentrations (15.9 vs. 15.4%, *p* = 1.000). In univariate Cox regression analysis, factors significantly associated with the development of composite CV outcome were history of hypertension (HR, 2.399; 95% CI, 1.257–4.579; *p* = 0.008), use of angiotensin-converting enzyme inhibitor (ACEi) or angiotensin II receptor blocker (ARB) (HR, 1.669; 95% CI, 1.007–2.765; *p* = 0.047), serum levels of hemoglobin (HR, 0.85; 95% CI, 0.737–0.982; *p* = 0.027), eGFR (HR, 0.99; 95% CI, 0.98–0.999; *p* = 0.031), and presence of CAD (HR, 3.45; 95% CI, 1.881–6.325; *p* ≤ 0.001), and Syntax score (HR, 1.033; 95% CI, 1.015–1.051). After performing multivariable forward-stepwise Cox regression analysis, history of hypertension (HR 1.983; 95% CI 1.033–3.806) and presence of CAD (HR 3.45; 95% CI 1.881–6.325) remained significantly associated with composite CV outcome, as shown in Table 2.

**Table 2:** Univariate and multivariate analyses of factors associated with composite cardiovascular outcome.

Variable	Univariate Cox regression			Multivariate Cox regression^a^		
	HR	95% CI	*P* value	HR	95% CI	*P* value
Age (years)	1.01	0.991–1.029	0.312			
Sex (male)	1.245	0.718–2.158	0.436			
Smoking	0.997	0.61–1.63	0.991			
BMI (kg/m^2^)	0.982	0.927–1.039	0.524			
Medical History						
Hypertension	2.399	1.257–4.579	0.008	1.983	1.033–3.806	0.04
Diabetes mellitus	1.345	0.830–2.182	0.229			
Heart failure	1.583	0.903–2.777	0.109			
Chronic kidney disease	1.506	0.904–2.508	0.116			
Medications						
Antiplatelet	1.117	0.683–1.826	0.659			
ACEi or ARB	1.669	1.007–2.765	0.047			
BB	1.603	0.95–2.707	0.077			
Statin	1.555	0.944–2.562	0.083			
Laboratory data						
Hemoglobin (g/dL)	0.85	0.737–0.982	0.027			
Fasting glucose (mg/dL)	1.004	0.999–1.009	0.144			
Low density lipoprotein (mg/dL)	0.997	0.989–1.005	0.491			
High density lipoprotein (mg/dL)	0.989	0.975–1.004	0.168			
eGFR (mL/min/1.73 m^2^)	0.99	0.98–0.999	0.031			
Uric acid (mg/dL)	1.062	0.928–1.215	0.382			
Proteinuria, n (%)	1.222	0.64–2.334	0.543			
Log PCSK6^b^	1.187	0.938–1.503	0.154			
Log Corin^b^	1.402	0.46–4.269	0.552			
Coronary angiography						
Coronary artery disease	3.45	1.881–6.325	<0.001	3.129	1.698–5.764	<0.001
Syntax score	1.033	1.015–1.051	<0.001			

BMI, body mass index; ACEi, angiotensin-converting enzyme inhibitor; ARB, angiotensin II receptor blocker; eGFR, estimated glomerular filtration rate

^a^The model consists of age, gender, and variables with *p* < 0.05 in univariate comparison

^b^Log transformation was performed before analysis

**Fig 1:**
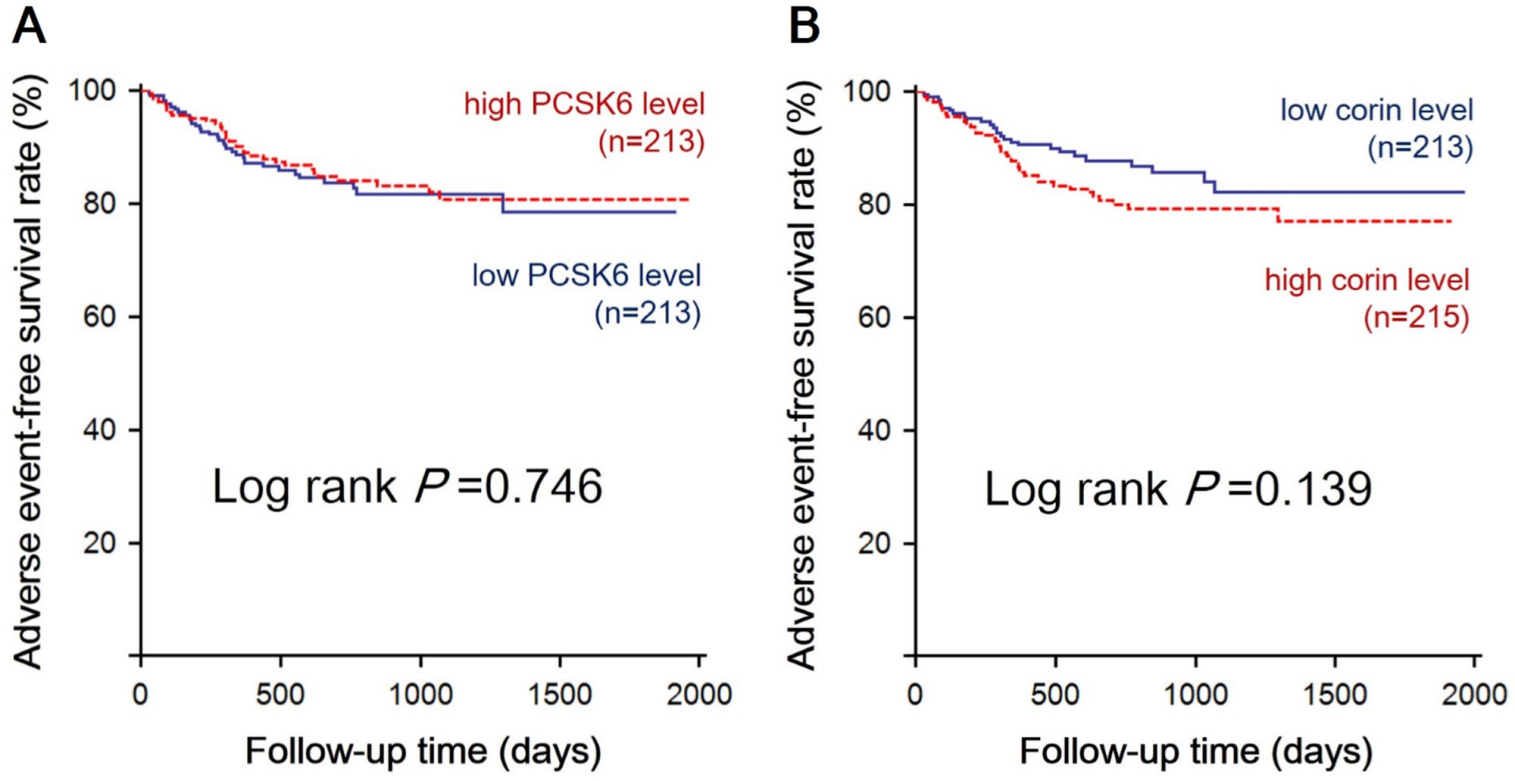
Kaplan–Meier estimate of composite cardiovascular outcomes in patients with various levels of serum PCSK6 and corin.

Kaplan–Meier survival analysis was performed to investigate the potential impact of baseline serum PCSK6 and corin levels on CV event-free survival. The risks of a composite CV event similar in patients with high vs. low PCSK6 and in patients with high vs. low corin levels (p = 0.746 and 0.139, respectively), as illustrated in Fig 1.

### Stratified analysis of serum PCSK6 and corin levels in predicting composite CV outcomes

The study cohort was stratified by gender and the presence of hypertension, diabetes, CAD, heart failure, and CKD. As shown in Table 3, there was no significant interaction between serum PCSK6 level and subgroup with respect to predicting composite CV events. After adjustment for significant risk factors identified in Cox’s regression model, the association did not reach statistical significance in the subsets of patients with different gender, or with high CV risk profiles, including hypertension, diabetes mellitus, CKD, heart failure, or CAD. However, an increased risk was seen in non-CKD patients with higher PCSK6 levels (adjusted HR 1.38; 95% CI, 1.023–1.862; *p* = 0.035).

**Table 3:** Stratified analysis of risk for composite cardiovascular outcome in patients grouped by gender and by the presence of diabetes, hypertension, CAD, heart failure, and/or chronic kidney disease.

Subgroup (events/ subjects)	Log PCSK6				Log corin			
	Crude HR (95% CI)	*P*	Adjusted HR (95% CI)*	*P*	Crude HR (95% CI)	*P*	Adjusted HR (95% CI)*	*P*
Overall (67 / 429)	1.187 (0.938–1.503)	0.154	1.225 (0.956–1.571)	0.109	1.402 (0.460–4.269)	0.552	1.366 (0.469–3.982)	0.568
Gender								
Male (50/299)	2.248 (0.851–1.522)	0.382	1.128 (0.830–1.532)	0.443	1.266 (0.328–4.885)	0.732	1.611 (0.468–5.547)	0.450
Female (17/130)	1.314 (0.864–1.999)	0.201	1.613 (0.980–2.654)	0.060	1.233 (0.134–11.311)	0.853	0.745 (0.077–7.210)	0.799
Hypertension								
Yes (56 / 299)	1.218 (0.92–1.612)	0.168	1.191 (0.889–1.597)	0.241	0.934 (0.303–2.878)	0.905	1.093 (0.371–3.224)	0.871
No (11 / 130)	1.232 (0.756–2.004)	0.403	1.309 (0.788–2.174)	0.298	6.219 (0.326–118.46)	0.224	16.122 (0.236–1101.1)	0.197
Diabetes mellitus								
Yes (29 / 162)	1.150 (0.794–1.664)	0.459	1.114 (0.741–1.675)	0.604	1.467 (0.331–6.500)	0.614	0.652 (0.160–2.653)	0.550
No (38 / 267)	1.206 (0.886–1.642)	0.234	1.243 (0.880–1.755)	0.217	1.319 (0.258–6.735)	0.740	1.348 (0.199–9.140)	0.760
CAD								
Yes (54 / 243)	1.145 (0.881–1.488)	0.311	1.175 (0.899–1.535)	0.239	1.063 (0.349–3.239)	0.915	1.113 (0.338–3.664)	0.860
No (13 / 186)	1.496 (0.838–2.670)	0.173	1.147 (0.510–2.579)	0.741	6.965 (0.323–150.37)	0.216	3.458 (0.099–120.27)	0.493
Heart failure								
Yes (16 / 76)	1.379 (0.816–2.332)	0.230	1.337 (0.707–2.531)	0.372	1.190 (0.193–7.335)	0.851	1.312 (0.305–5.644)	0.715
No (51 / 353)	1.154 (0.884–1.506)	0.292	1.215 (0.929–1.590)	0.155	1.842 (0.462–7.348)	0.387	1.341 (0.285–6.321)	0.711
CKD								
Yes (22 / 117)	0.959 (0.609–1.510)	0.855	0.917 (0.539–1.559)	0.749	0.458 (0.130–1.619)	0.225	0.466 (0.113–1.916)	0.290
No (45 / 312)	1.303 (0.981–1.730)	0.067	1.380 (1.023–1.862)	0.035	5.123 (1.003–26.164)	0.050	4.407 (0.686–28.304)	0.118

CAD, coronary artery disease; CKD, chronic kidney disease.

*adjusted for age, gender, hypertension, ACE-I/ARB, BB, hemoglobin, eGFR, coronary artery disease, Syntax score

### Correlation between serum PCSK6 and corin levels

**Fig 2:**
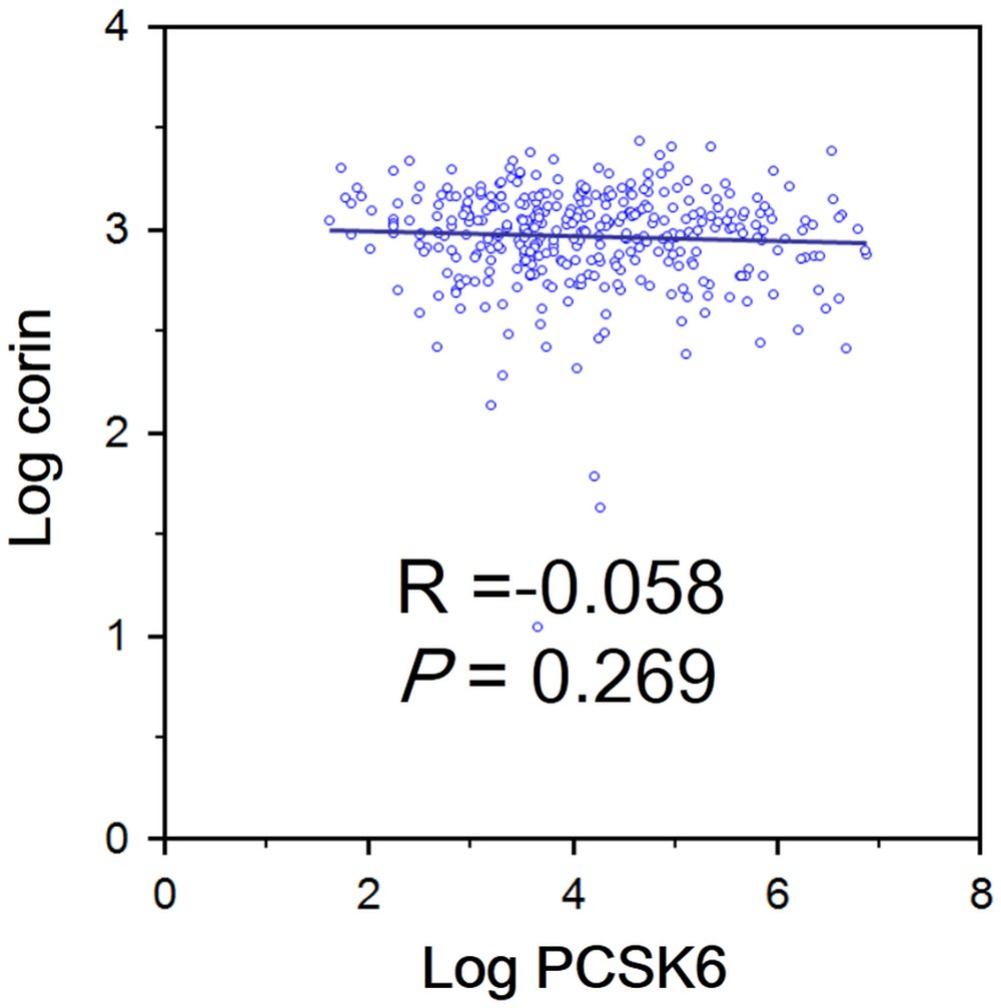
The association between serum PCSK6 and serum corin levels.

Linear regression analysis was performed to investigate the correlation between serum PCSK6 and corin levels. Pearson’s test showed an insignificant association between log serum PCSK6 and log serum corin levels (*p* = 0.269), as shown in Fig 2.

## Discussion

The major findings of the present study are that baseline serum PCSK6 and corin levels are not associated with the risk for a composite CV outcome in patients undergoing coronary angiography. The association remained insignificant in patients with different gender, or with higher cardiovascular risk, including those with hypertension (HTN), diabetes mellitus (DM), CAD, heart failure, and/or CKD. However, in non-CKD patients, higher serum PCSK6 may indicate increased risk for CV events after coronary angiography. To the best of our knowledge, the present study is the first to evaluate the association between serum PCSK6 level and CV outcomes. Our results suggest that higher serum PCSK6 levels may be a marker for higher CV risk in non-CKD patients.

PCSK6 is one of the protein convertases that activates precursor proteins by cleaving paired basic residues [11]. PCSK6 is widely expressed in many tissues and is secreted via the trans-Golgi network [11]. It is activated at the cell surface and the extracellular matrix, where it binds to heparin sulfate proteoglycans [11, 20]. The Nodal and Lefty system is cleaved by secreted PCSK6 during the development, and single-nucleotide polymorphisms of PCSK6 have been associated with left handedness and/or dyslexia [21, 22]. Other known substrates of PCSK6 including metalloproteinases, angiopoietin-like 3, Vpr (an HIV-1 accessory protein), membrane transferrin receptor-2, and preprohepcidin [11, 23, 24].

PCSK6 was recently found to activate corin in the heart [9]. Corin is primarily expressed in ANP-expressing atrial cardiomyocytes and acts principally to convert pro-ANP to ANP [1]. Previous studies showed that cardiac corin expression is stimulated in the context of pathological cardiac remodeling. Decreased corin activity is associated with worse cardiovascular outcomes [6–8, 13, 25, 26]. These results indicate that activation of the PCSK6-corin-ANP pathway plays a protective role in cardiac remodeling.

After activation at the cell surface, corin is released to the circulation after autocleavage or proteolysis mediated by adisintegrin and metalloprotease (ADMA). Serum corin level may therefore represent a marker of cardiac corin activity [13]. Previous studies showed that serum corin may predict CV outcomes in patients with cardiovascular diseases, such as hypertension, heart failure, myocardial infarction, and stroke [14–17]. However, the role of serum PCSK6 levels in predicting CV outcomes has not previously been evaluated.

In this study, we evaluated patients admitted for scheduled coronary angiography; 56.8% of all participants had confirmed CAD. After a median follow-up of 691 days, pre-procedural serum levels of neither PCSK6 nor corin predicted cardiovascular events. This lack of a correlation between serum levels of PCSK6 and corin suggests that circulating PCSK6 and corin may differ in terms of secretion site and/or regulatory mechanisms.

One possible explanation for this negative result is that serum levels of PCSK6 and corin are not an accurate index for levels of cardiac PCSK6/corin/ANP activity. PCSK6 is widely expressed, and secretion from cardiac cells may not correlate with circulatory level. Although the level of serum corin has been associated with several CV outcomes in patients with various CV diseases, previous studies have shown that the level of circulating corin does not reflect the level of corin in tissue [27]. Notably, some circulating corin is enzymatically inactive [2]. Previous clinical studies examined only the level of circulating corin, rather than the activity of this circulating corin. The precise mechanisms underlying the relationship between circulating soluble corin and CV disease remain unclear.

Another explanation for this negative result may be the relatively mild CV disease burden in our cohort. A role for serum corin in predicting CV outcomes has been reported in prospective studies that included patients with acute coronary syndrome, acute myocardial infarction, heart failure, and/or acute stroke [14–17]. Another study showed that a larger decrease in plasma corin concentration after coronary artery bypass surgery is associated with increased risk for heart failure [28]. The patients included in our cohort were in relatively stable condition; only 76 participants (17.7%) had chronic heart failure. Sixty-seven participants (14.7%) had no CV disease. At the time of patient enrollment in the study, we noted no instance of acute CV stress, such as acute coronary syndrome or acute stroke, which may have limited the occurrence of CV outcomes during follow-up. In the subgroup analysis, the predictive roles of circulatory PCSK6 and corin remained insignificant in participants with increased CV risk, including those with DM, HTN, heart failure, and/or CKD. However, a significant association was noted between serum PCSK6 level and CV events in non-CKD patients. The impact of renal function on the level of circulatory PCSK6 remains unclear, and more data are needed to confirm this finding.

The final explanation for this negative result may be that the CV event encountered most frequently in this study was coronary revascularization (51 of 67 events, 76.1%). A previous study reported corin expression in mouse aorta; expression levels were elevated in a mouse model of atherosclerosis [29]. However, in a large prospective study of AMI patients, Zhou et al. showed that serum corin was not a predictor of recurrent AMI [15]. Our result supports the idea that the PCSK6/corin/ANP pathway may not play a role in the progression of atherosclerosis. However, conflicting data have been reported in the context of non-ST segment elevation myocardial infarction (non-STEMI) patients [17]. Additional studies will be necessary for clarification.

This study had some limitations. First, the study population was relatively small. All study participants were of Asian ethnicity and were recruited from a single tertiary center. Second, the levels of serum PCSK6 and corin were measured on the date of coronary angiography. We were not able to evaluate the predicting value of the changes of these biomarkers. Third, we were not able to distinguish different corin isoforms in the blood samples. Whether the composition of various corin isoforms has prognostic needs further evaluation. Fourth, it was not possible to measure levels of other relevant biomarkers (e.g., pro-ANP, NT-pro-BNP). It was also not possible to measure activity levels of serum PCSK6 or corin. Thus, we could not explore potential mechanisms underlying the association between the PCSK/corin/ANP pathway and progressive CV disease. Finally, our patients had relatively mild CV burden with only 57% of them had confirmed CAD. Besides, most of the CV events experienced by members of the patient cohort involved coronary revascularization (51 out of 75 events). Other CV events observed included acute stroke (2 events), AMI (7 events), and CV-related death (15 events). However, the low number of events reported hindered further analysis of the predictive value of PCSK6 and corin. Our study demonstrated that serum PCSK6 and corin levels are not risk markers for CV outcomes in patients with suspected CAD.

## Conclusion

Our data showed that baseline serum PCSK6 and corin levels are not independent prognostic markers for CV outcomes in patients undergoing coronary angiography. However, higher serum PCSK6 levels may indicate increased risk for CV events in non-CKD patients. Further studies are needed to confirm our findings and to clarify the role of the PCSK6/corin/ANP pathway in the progression of CV disease.

## Supporting information

S1 Fig The association between (A) serum PCSK6 and (B) serum corin levels. (TIF)

S1 Table Univariate and multivariate linear regression analysis for predictors of serum PSCK6 levels. (DOCX)

S2 Table Baseline patient characteristics, stratified by left ventricular ejection fraction. (DOCX)

S3 Table Univariate and multivariate analyses of factors associated with composite cardiovascular outcome in patients with left ventricular ejection fraction less than 40%. (DOCX)

S4 Table Baseline patient characteristics, stratified by gender. (DOCX)

S5 Table Baseline patient characteristics, stratified by chronic kidney disease status. (DOCX)
