# Supplementary figures and images for: Serum PCSK6 and corin levels are not associated with cardiovascular outcomes in patients undergoing coronary angiography
Source: PLoS One. 2019 Dec 11;14(12):e0226129. doi: 10.1371/journal.pone.0226129 (PMC6905542; doi:10.1371/journal.pone.0226129)

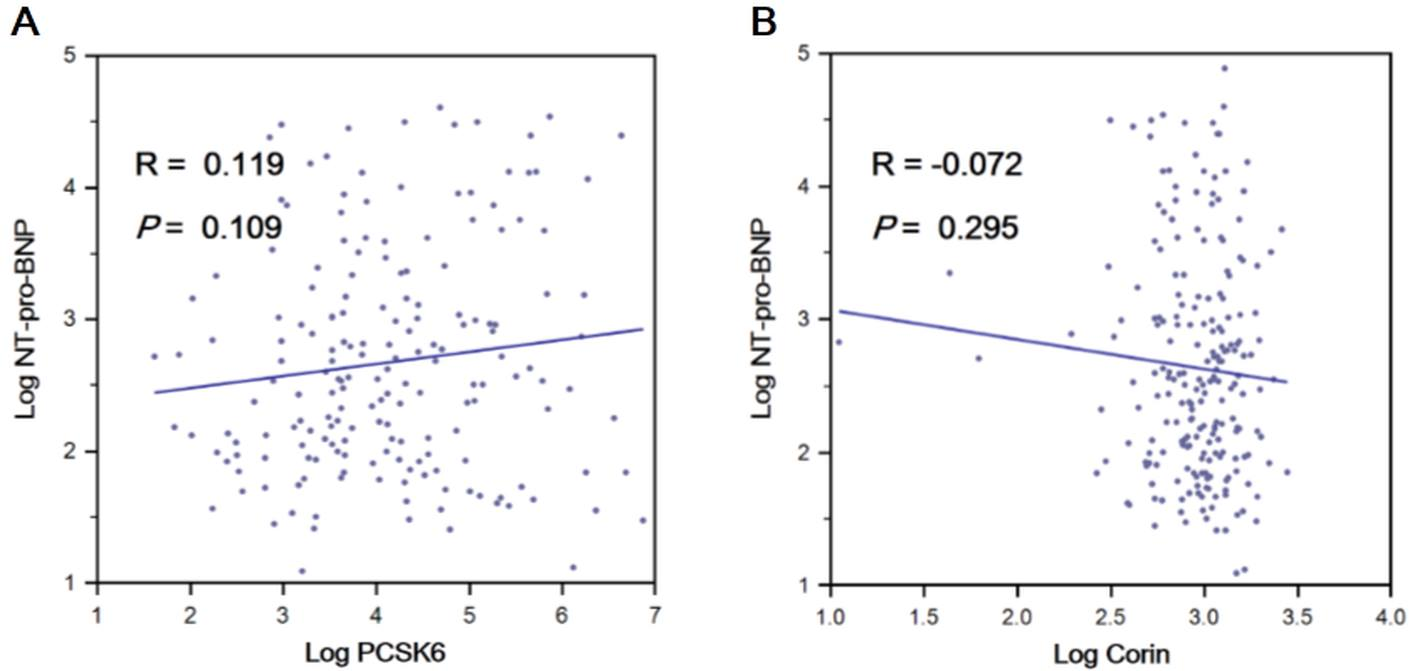

Supplement: S1 Fig — (TIF) [file pone.0226129.s001.tif]
