# Supplementary material for: Serum PCSK6 and corin levels are not associated with cardiovascular outcomes in patients undergoing coronary angiography
Source: PLoS One. 2019 Dec 11;14(12):e0226129. doi: 10.1371/journal.pone.0226129 (PMC6905542; doi:10.1371/journal.pone.0226129)
Supplement: S1 Table — (DOCX) [file pone.0226129.s002.docx]

S1 Table. Univariate and multivariate linear regression analysis for predictors of serum PSCK6 levels

|  | **Univariate linear regression** | | | **Multivariate linear regression^a^** | | |
| --- | --- | --- | --- | --- | --- | --- |
| Variable | β | 95% CI | *P* value | β | 95% CI | *P* value |
| Age (years) | -0.135 | -0.020 - -0.003 | 0.010 | -0.127 | -0.020 – -0.002 | 0.015 |
| Sex (male) | -0.129 | -0.561 - -0.063 | 0.014 | -0.120 | -0.539 – -0.043 | 0.022 |
| Smoking | -0.028 | -0.305 – 0.175 | 0.597 |  |  |  |
| BMI (kg/m^2^) | 0.002 | -0.027 – 0.028 | 0.972 |  |  |  |
| Medical History |  |  |  |  |  |  |
| Hypertension | -0.083 | -0.460 – 0.049 | 0.114 |  |  |  |
| Diabetes mellitus | -0.003 | -0.248 – 0.236 | 0.959 |  |  |  |
| Heart failure | -0.019 | -0.353 – 0.245 | 0.721 |  |  |  |
| Chronic kidney disease | 0.002 | -0.255 – 0.263 | 0.975 |  |  |  |
| Medications |  |  |  |  |  |  |
| Antiplatelet | -0.116 | -0.501 - -0.029 | 0.028 |  |  |  |
| ACEi or ARB | -0.024 | -0.333 – 0.206 | 0.643 |  |  |  |
| BB | -0.030 | -0.367 – 0.203 | 0.572 |  |  |  |
| Statin | -0.066 | -0.427 – 0.096 | 0.214 |  |  |  |
| Laboratory data |  |  |  |  |  |  |
| Hemoglobin (g/dL) | -0.024 | -0.087 – 0.054 | 0.645 |  |  |  |
| Fasting glucose (mg/dL) | -0.042 | -0.005 – 0.002 | 0.431 |  |  |  |
| Low density lipoprotein (mg/dL) | 0.075 | -0.001 – 0.007 | 0.153 |  |  |  |
| High density lipoprotein (mg/dL) | -0.004 | -0.007 – 0.007 | 0.938 |  |  |  |
| eGFR (mL/min/1.73 m^2^) | 0.053 | -0.002 – 0.007 | 0.314 |  |  |  |
| Uric acid (mg/dL) | 0.028 | -0.047 – 0.082 | 0.590 |  |  |  |
| Proteinuria, n (%) | 0.038 | -0.207 – 0,448 | 0.470 |  |  |  |
| Log Corin | -0.058 | -0.732 – 0.205 | 0.269 |  |  |  |
| Coronary angiography |  |  |  |  |  |  |
| Coronary artery disease | -0.030 | -0.306 – 0.169 | 0.572 |  |  |  |
| Syntax score | -0.039 | -0.014 – 0.006 | 0.461 |  |  |  |

BMI, body mass index; ACEi, angiotensin-converting enzyme inhibitor; ARB, angiotensin II receptor blocker; eGFR, estimated glomerular filtration rate

^a^The model consists of age, gender, and variables with *p* < 0.05 in univariate comparison
