# Supplementary material for: Serum PCSK6 and corin levels are not associated with cardiovascular outcomes in patients undergoing coronary angiography
Source: PLoS One. 2019 Dec 11;14(12):e0226129. doi: 10.1371/journal.pone.0226129 (PMC6905542; doi:10.1371/journal.pone.0226129)
Supplement: S2 Table — (DOCX) [file pone.0226129.s003.docx]

S2 Table. Baseline patient characteristics, stratified by left ventricular ejection fraction

|  | **LVEF <=40%** | **LVEF >40%** |  |
| --- | --- | --- | --- |
| Characteristic | n = 29 | n = 341 | *P* value |
| Age (years) | 75 (61-84.5) | 71 (60-80) | 0.134 |
| Sex (male) | 25 (86.2) | 228 (66.9) | 0.036 |
| Smoking | 13 (44.8) | 119 (34.9) | 0.315 |
| BMI (kg/m^2^) | 23.3 (22.0-25.4) | 25.6 (23.3-28.1) | 0.003 |
| Medical History |  |  |  |
| Hypertension | 20 (69) | 245 (71.8) | 0.830 |
| Diabetes mellitus | 12 (41.4) | 120 (35.2) | 0.547 |
| Heart failure | 29 (100) | 40 (11.7) | <0.001 |
| Chronic kidney disease | 17 (58.6) | 78 (22.9) | <0.001 |
| Medications |  |  |  |
| Antiplatelet | 15 (51.7) | 204 (59.8) | 0.434 |
| ACEi or ARB | 6 (20.7) | 92 (27.0) | 0.520 |
| BB | 3 (10.3) | 80 (23.5) | 0.161 |
| Statin | 4 (13.8) | 97 (28.4) | 0.127 |
| Laboratory data |  |  |  |
| Hemoglobin (g/dL) | 13 (11.3-14) | 13 (11.8-13.9) | 0.765 |
| Fasting glucose (mg/dL) | 115.9 (95-145) | 104 (93-120) | 0.161 |
| Low density lipoprotein (mg/dL) | 84 (64-101.5) | 96.5 (75-113) | 0.060 |
| High density lipoprotein (mg/dL) | 40 (30.1-48.2) | 41.6 (32-54.3) | 0.162 |
| eGFR (mL/min/1.73 m^2^) | 53.8 (37.2-85.5) | 77.9 (62.2-93.8) | 0.004 |
| Uric acid (mg/dL) | 7 (5.9-8.2) | 6.1 (4.8-7.1) | 0.001 |
| Proteinuria, n (%) | 10 (34.5) | 41 (12) | 0.003 |
| PCSK6 (ng/mL) | 67.3 (37.8-176.6) | 57.9 (27.7-138.6) | 0.324 |
| Corin (pg/mL) | 942.9 (695.3-1297.5) | 1054.2 (755.5-1329.4) | 0.681 |
| Coronary angiography |  |  |  |
| Coronary artery disease | 18 (62.1) | 200 (58.7) | 0.845 |
| Syntax score | 10 (0-34.5) | 3 (0-12) | 0.040 |

Data are presented as median (interquartile range) or as total number of patients (%).

LVEF, left ventricular ejection fraction; BMI, body mass index; ACEi, angiotensin-converting enzyme inhibitor; ARB, angiotensin II receptor blocker; eGFR, estimated glomerular filtration rate
