# Supplementary material for: Serum PCSK6 and corin levels are not associated with cardiovascular outcomes in patients undergoing coronary angiography
Source: PLoS One. 2019 Dec 11;14(12):e0226129. doi: 10.1371/journal.pone.0226129 (PMC6905542; doi:10.1371/journal.pone.0226129)
Supplement: S3 Table — (DOCX) [file pone.0226129.s004.docx]

S3 Table. Univariate and multivariate analyses of factors associated with composite cardiovascular outcome in patients with left ventricular ejection fraction less than 40%

|  | **Univariate Cox regression** | | |
| --- | --- | --- | --- |
| Variable | HR | 95% CI | *P* value |
| Age (years) | 1.001 | 0.946-1.058 | 0.983 |
| Sex (male) | 22.9 | 0-71827111 | 0.681 |
| Smoking | 0.350 | 0.039-3.139 | 0.348 |
| BMI (kg/m^2^) | 0.988 | 0.803-1.217 | 0.911 |
| Medical History |  |  |  |
| Hypertension | 1.499 | 0.167-13.419 | 0.717 |
| Diabetes mellitus | 0.407 | 0.045-3.644 | 0.422 |
| Chronic kidney disease | 1.215 | 0.203-7.282 | 0.831 |
| Medications |  |  |  |
| Antiplatelet | 0.273 | 0.031-2.444 | 0.246 |
| ACEi or ARB | 0.968 | 0.108-8.672 | 0.976 |
| BB | 0.041 | 0.000-8587 | 0.610 |
| Statin | 1.523 | 0.170-13.677 | 0.707 |
| Laboratory data |  |  |  |
| Hemoglobin (g/dL) | 0.796 | 0.521-1.216 | 0.291 |
| Fasting glucose (mg/dL) | 1.011 | 0.992-1.030 | 0.268 |
| Low density lipoprotein (mg/dL) | 1.002 | 0.970-1.037 | 0.885 |
| High density lipoprotein (mg/dL) | 0.924 | 0.842-1.014 | 0.096 |
| eGFR (mL/min/1.73 m^2^) | 0.989 | 0.959-1.020 | 0.488 |
| Uric acid (mg/dL) | 1.181 | 0.882-1.582 | 0.264 |
| Proteinuria, n (%) | 3.071 | 0.509-18.553 | 0.221 |
| Log PCSK6 | 4.000 | 0.617-25.927 | 0.146 |
| Log Corin | 10.39 | 0.167-644.61 | 0.266 |
| Coronary angiography |  |  |  |
| Coronary artery disease | 1.118 | 0.186-6.709 | 0.903 |
| Syntax score | 0.956 | 0.885-1.033 | 0.252 |

BMI, body mass index; ACEi, angiotensin-converting enzyme inhibitor; ARB, angiotensin II receptor blocker; eGFR, estimated glomerular filtration rate
