# Supplementary material for: Serum PCSK6 and corin levels are not associated with cardiovascular outcomes in patients undergoing coronary angiography
Source: PLoS One. 2019 Dec 11;14(12):e0226129. doi: 10.1371/journal.pone.0226129 (PMC6905542; doi:10.1371/journal.pone.0226129)
Supplement: S4 Table — (DOCX) [file pone.0226129.s005.docx]

S4 Table. Baseline patient characteristics, stratified by gender

|  | **Male** | **Female** |  |
| --- | --- | --- | --- |
| Characteristic | n = 299 | n = 130 | *P* value |
| Age (years) | 72 (59-82) | 70 (61-76) | 0.089 |
| Smoking | 149 (49.8) | 16 (12.3) | <0.001 |
| BMI (kg/m^2^) | 25.4 (23.2-27.7) | 25.5 (22.8-28.6) | 0.747 |
| Medical History |  |  |  |
| Hypertension | 216 (72.2) | 83 (63.8) | 0.087 |
| Diabetes mellitus | 112 (37.5) | 50 (38.5) | 0.914 |
| Heart failure | 55 (18.4) | 21 (16.2) | 0.680 |
| Chronic kidney disease | 83 (27.8) | 34 (26.2) | 0.814 |
| Medications |  |  |  |
| Antiplatelet | 182 (60.9) | 70 (53.8) | 0.200 |
| ACEi or ARB | 73 (24.4) | 35 (26.9) | 0.629 |
| BB | 64 (21.4) | 30 (23.1) | 0.705 |
| Statin | 79 (26.4) | 35 (26.9) | 0.906 |
| Laboratory data |  |  |  |
| Hemoglobin (g/dL) | 13.3 (11.9-14.2) | 12.3 (11.1-13.1) | <0.001 |
| Fasting glucose (mg/dL) | 106 (93-126) | 101.5 (91-120.3) | 0.385 |
| Low density lipoprotein (mg/dL) | 93 (75-110) | 97.5 (83-115.3) | 0.017 |
| High density lipoprotein (mg/dL) | 37.8 (30-49.6) | 46.9 (38-62.4) | <0.001 |
| eGFR (mL/min/1.73 m^2^) | 74.5 (57.2-89.4) | 79.7 (58.4-97.6) | 0.113 |
| Uric acid (mg/dL) | 6.1 (5.1-7.3) | 5.6 (4.3-6.5) | <0.001 |
| Proteinuria, n (%) | 42 (14) | 18 (13.8) | 1.000 |
| PCSK6 (ng/mL) | 51.1 (26.2-119.8) | 70.9 (34.1-199.9) | 0.018 |
| Corin (pg/mL) | 1161.4 (893.9-1471) | 806.4 (565.1-1038.4) | <0.001 |
| Coronary angiography |  |  |  |
| Coronary artery disease | 181 (60.5) | 62 (47.7) | 0.015 |
| Syntax score | 5 (0-15) | 0 (0-11.3) | 0.006 |

Data are presented as median (interquartile range) or as total number of patients (%).

BMI, body mass index; ACEi, angiotensin-converting enzyme inhibitor; ARB, angiotensin II receptor blocker; eGFR, estimated glomerular filtration rate
