# Supplementary material for: Serum PCSK6 and corin levels are not associated with cardiovascular outcomes in patients undergoing coronary angiography
Source: PLoS One. 2019 Dec 11;14(12):e0226129. doi: 10.1371/journal.pone.0226129 (PMC6905542; doi:10.1371/journal.pone.0226129)
Supplement: S5 Table — (DOCX) [file pone.0226129.s006.docx]

S5 Table. Baseline patient characteristics, stratified by chronic kidney disease status

|  | **CKD** | **non-CKD** |  |
| --- | --- | --- | --- |
| Characteristic | n = 117 | n = 312 | *P* value |
| Age (years) | 78 (70-83) | 67 (58-79) | <0.001 |
| Sex (male) | 83 (70.9) | 216 (69.2) | 0.814 |
| Smoking | 43 (36.8) | 122 (39.1) | 0.728 |
| BMI (kg/m^2^) | 25.5 (23-27.5) | 25.4 (23.1-28) | 0.691 |
| Medical History |  |  |  |
| Hypertension | 99 (84.6) | 200 (64.1) | <0.001 |
| Diabetes mellitus | 69 (59) | 93 (29.8) | <0.001 |
| Heart failure | 32 (27.4) | 44 (14.1) | 0.003 |
| Medications |  |  |  |
| Antiplatelet | 67 (57.3) | 185 (59.3) | 0.742 |
| ACEi or ARB | 41 (35) | 67 (21.5) | 0.006 |
| BB | 34 (29.1) | 60 (19.2) | 0.036 |
| Statin | 29 (24.8) | 85 (27.2) | 0.713 |
| Laboratory data |  |  |  |
| Hemoglobin (g/dL) | 12.1 (10.6-13.4) | 13.1 (11.9-14) | <0.001 |
| Fasting glucose (mg/dL) | 109 (95.5-146.5) | 102.5 (91-119.8) | 0.005 |
| Low density lipoprotein (mg/dL) | 94 (72-109.5) | 96.5 (78.3-114) | 0.184 |
| High density lipoprotein (mg/dL) | 41.6 (32.1-51.3) | 41.9 (31.2-54.2) | 0.972 |
| eGFR (mL/min/1.73 m^2^) | 46.9 (36.2-53.7) | 83.7 (73.7-98.5) | <0.001 |
| Uric acid (mg/dL) | 6.7 (5.7-8) | 5.8 (4.6-6.8) | <0.001 |
| Proteinuria, n (%) | 43 (36.8) | 17 (5.4) | <0.001 |
| PCSK6 (ng/mL) | 55.3 (29.7-126) | 49.5 (25.8-143) | 0.975 |
| Corin (pg/mL) | 978.5 (716.9-1423.4) | 1055.8 (754.4-1359.2) | 0.668 |
| Coronary angiography |  |  |  |
| Coronary artery disease | 66 (56.4) | 177 (56.7) | 1.000 |
| Syntax score | 4 (0-13.5) | 3 (0-14) | 0.759 |

Data are presented as median (interquartile range) or as total number of patients (%).

CKD, chronic kidney disease; BMI, body mass index; ACEi, angiotensin-converting enzyme inhibitor; ARB, angiotensin II receptor blocker; eGFR, estimated glomerular filtration rate
